# Circadian deep sequencing reveals stress-response genes that adopt robust rhythmic expression during aging

**DOI:** 10.1038/ncomms14529

**Published:** 2017-02-21

**Authors:** Rachael C. Kuintzle, Eileen S. Chow, Tara N. Westby, Barbara O. Gvakharia, Jadwiga M. Giebultowicz, David A Hendrix

**Affiliations:** 1 Department of Biochemistry & Biophysics, 2011 Agriculture & Life Sciences Building, Oregon State University, Corvallis, Oregon 97331, USA; 2 Department of Integrative Biology, 3029 Cordley Hall, Oregon State University, Corvallis, Oregon 97331, USA; 3 School of Electrical Engineering and Computer Science, 1148 Kelley Engineering Center, Oregon State University, Corvallis, Oregon 97331, USA

## Abstract

Disruption of the circadian clock, which directs rhythmic expression of numerous output genes, accelerates aging. To enquire how the circadian system protects aging organisms, here we compare circadian transcriptomes in heads of young and old *Drosophila melanogaster*. The core clock and most output genes remained robustly rhythmic in old flies, while others lost rhythmicity with age, resulting in constitutive over- or under-expression. Unexpectedly, we identify a subset of genes that adopted increased or *de novo* rhythmicity during aging, enriched for stress-response functions. These genes, termed late-life cyclers, were also rhythmically induced in young flies by constant exposure to exogenous oxidative stress, and this upregulation is CLOCK-dependent. We also identify age-onset rhythmicity in several putative primary piRNA transcripts overlapping antisense transposons. Our results suggest that, as organisms age, the circadian system shifts greater regulatory priority to the mitigation of accumulating cellular stress.

## Abstract (web summary)

Disruption of circadian rhythms leads to reduced healthspan, but the mechanisms by which the normal clock protects aging organisms are not known. Here, the authors show that a subset of genes becomes more rhythmically expressed in older flies, and these are enriched for response to oxidative stress.

The circadian system confers integrated temporal control to organismal processes at the molecular, cellular, physiological and behavioural levels. Cellular clocks are based on negative feedback loops driven by core clock genes, which oscillate with ∼24 h periodicity and are largely conserved from flies to mammals^1^. In *Drosophila*, two transcription factors encoded by the genes *Clock* (*Clk*) and *cycle* (*cyc*) form the positive arm of the clock. Active CLK-CYC heterodimers stimulate transcription of target genes including the negative clock components encoded by *period* (*per*) and *timeless* (*tim*), which repress CLK-CYC activity. Entrainment of clock oscillations to LD cycles involves the photoreceptive protein CRY, which initiates degradation of TIM (ref. 1). Oscillating clock components impose rhythmic expression on a diverse portfolio of target clock-controlled genes (CCGs) that continues to expand as sensitivity of detection techniques improve^2^,^3^,^4^,^5^,^6^. These CCGs ultimately generate molecular and cellular rhythms, which profoundly influence metabolism and tissue homoeostasis. Disruption of the clock is associated with accelerated aging in mice, manifested in metabolic dysregulation^7^ and impairments of learning and memory^8^. In addition, the core molecular oscillator plays a conserved role in regulating the response to oxidative stress^9^,^10^,^11^, which increases during aging^12^ and contributes to age-related pathologies^13^. Accordingly, clock disruption also leads to increased oxidative damage and susceptibility to neurodegeneration in brains of aging *Drosophila*^14^,^15^ and mice^16^,^17^.

Despite substantial evidence that circadian disruption shortens healthspan, the mechanisms by which the clock protects aging organisms are not understood. To address this question, here we conduct an RNA sequencing (RNA-seq) study and compared diurnal expression of clock genes and CCGs in heads of young and old female flies. We detect dynamic reprogramming of the circadian transcriptome with some CCGs showing dampened or abolished oscillations in old flies. Surprisingly, a large subset of CCGs showed significantly larger expression amplitudes or *de novo* rhythmicity in old flies. Stress-response genes were enriched among transcripts with age-induced rhythmicity, hereafter called ‘late-life cyclers' (LLCs), and we show that exogenous oxidative stress can induce rhythmic LLC expression in young flies. Our data suggest that LLC activation is a strategy by which the clock helps organisms adapt to their changing cellular environments during aging.

## Results

### Pervasive alterations in rhythmic gene expression

To identify age-dependent changes in diurnal gene expression, we performed RNA-seq with RNA from heads of 5- or 55-day-old *w*^*1118*^ (*w*) females collected every 4 h for one daily cycle in two biological replicates in light-dark (LD) 12:12 h. Gene expression, quantified in units of Fragments Per Kilobase of transcript per Million mapped reads (FPKM) using the Tuxedo suite^18^,^19^ (Supplementary Table 1), showed high correlations between biological replicates in both young and old flies (Supplementary Fig. 1; Supplementary Table 2). Using ARSER (ref. 20), we identified 2,036 genes to be rhythmic in young flies, including 67% of genes previously deemed rhythmic at the messenger RNA (mRNA) level in heads of male flies^3^; this overlap is substantial considering differences in sex and periodicity detection methods (Methods). ARSER classified 1,887 genes to be rhythmic in old, including 922 that were also rhythmic in young (Supplementary Data 1).

**Figure 1:**
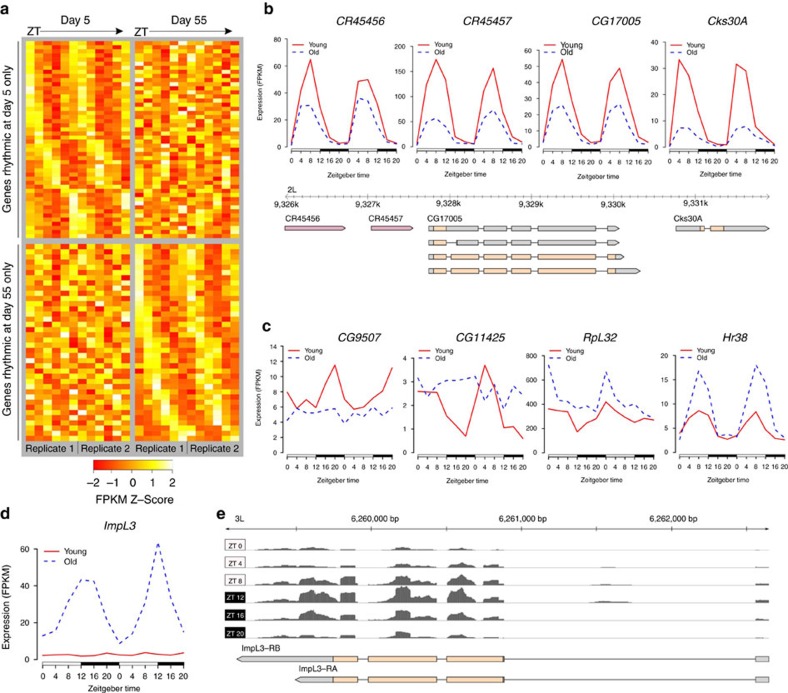
RNA-seq reveals diverse classes of age-dependent rhythmicity changes. (**a**) Heat map representing loss or gain of gene expression rhythmicity with age. Each column represents a single-time-point for a single-replicate sampled at 4 h intervals starting at Zeitgeber Time (ZT) 0. Hue represents the Z-score (FPKM minus mean over s.d.) of each gene (row) at each time point (column) for a given biological replicate. Genes in the top half are rhythmic in young and arrhythmic in old; those in the bottom are rhythmic in old and arrhythmic in young (Methods). (**b**–**d**) RNA-seq gene expression profiles; each period of ZT 0–20 represents a distinct biological replicate. (**b**) Coordinated, reduced amplitude in expression of circadian genes at the *Cks30A* gene locus. (**c**) Examples of genes rhythmic only in young (*CG9507* and *CG11425*); rhythmic in both young and old, with age-dependent increases in amplitude (*RpL32* and *Hr38*). (**d**,**e**) *ImpL3* adopts *de novo* rhythmic expression in old flies according to RNA-seq. Browser tracks in **e** show RNA-seq read pileups at each time point; reads represent merged RNA-seq data from replicate cohorts of old flies with the same y-axis range for all time points.

Although many CCGs maintained similar rhythmicity patterns throughout aging, a substantial number of genes showed marked changes in phase, amplitude or statistical rhythmic status. For example, 297 genes rhythmic in both young and old flies exhibited a ≥2 h shift in their time of peak expression (Supplementary Fig. 2). Further, 48 genes were highly rhythmic in young flies but arrhythmic in old, while 38 genes were highly rhythmic in old and arrhythmic in young flies (Fig. 1a, Methods). Examples of other age-related expression changes are shown in Fig. 1b–e. RNA-seq expression plots for all FlyBase genes and isoforms are available at: http://hendrixlab.cgrb.oregonstate.edu/youngAndOldExpression.html.

**Figure 2:**
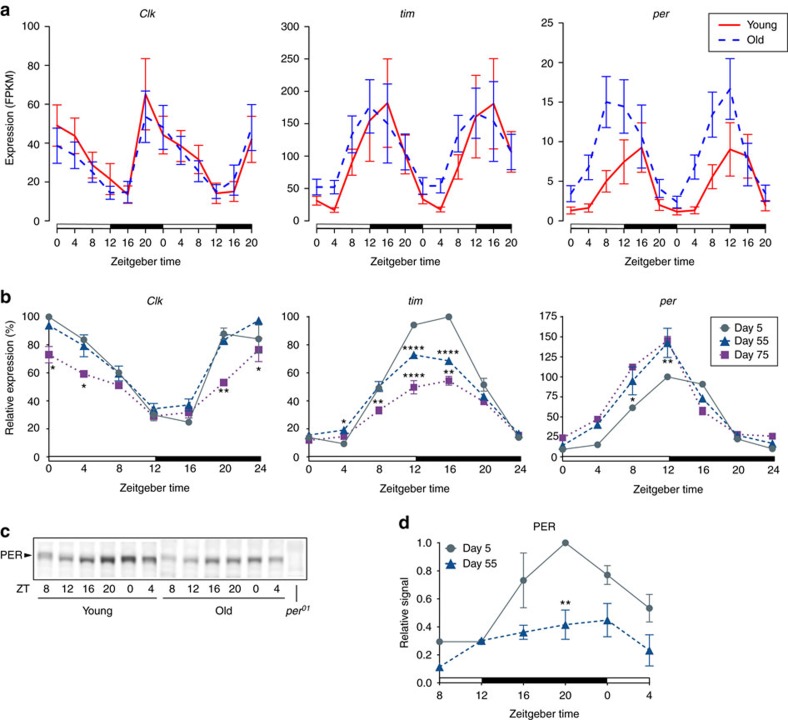
Core clock gene expression in young and old flies. (**a**) Age-dependent expression of three clock genes (RNA-seq). Each period of ZT 0–20 represents a distinct biological replicate. Error bars represent 95% confidence intervals reported by Cuffdiff. (**b**) qRT-PCR expression of *per*, *tim* and *Clk* in heads of females aged to 5, 55 and 75 days. Expression is reported as per cent of peak expression in day 5 flies. Data are mean (from two cycles)±s.e.m. (**c**) PER western blot from heads of young or old *w* flies collected at indicated ZT. The arrow indicates PER protein, with estimated molecular weight of 127-130 kDA, depending on the phosphorylation status. (**d**) PER levels (mean±s.e.m.) from western blot in **c**; *n*=3 biological replicates. (**b**,**c**) *P≤0.05; **P≤0.01; ***P≤0.001; ****P≤0.0001 (two-way ANOVA with Bonferroni correction; day 55 change is relative to day 5 values, and day 75 change is relative to day 55 values).

In accordance with the diversity of alterations in CCG expression, expression of some core clock genes also showed significant changes. Although *tim* expression remained highly rhythmic throughout aging, it showed a decline in peak expression, in agreement with previous qRT-PCR studies in heads of male and female flies^21^,^22^. *Clk* levels were highly rhythmic and not significantly reduced by day 55 (Fig. 2a); however, qRT-PCR with RNA from 75-day-old flies corroborated prior reports of a decline in *Clk* levels in very old age^21^ (Fig. 2b). In contrast with prior qPCR data^21^,^22^, RNA-seq revealed *per* peak expression to increase with age, and this was confirmed by qPCR (Fig. 2b). However, PER protein levels decreased significantly by day 55 (Fig. 2c,d; Supplementary Fig. 3), consistent with previous reports^21^,^22^. Thus, the opposite age-dependent changes in *per* and *tim* RNA levels suggest that the core circadian mechanism known in young flies may be altered during aging.

### Late-life cyclers

**Figure 3:**
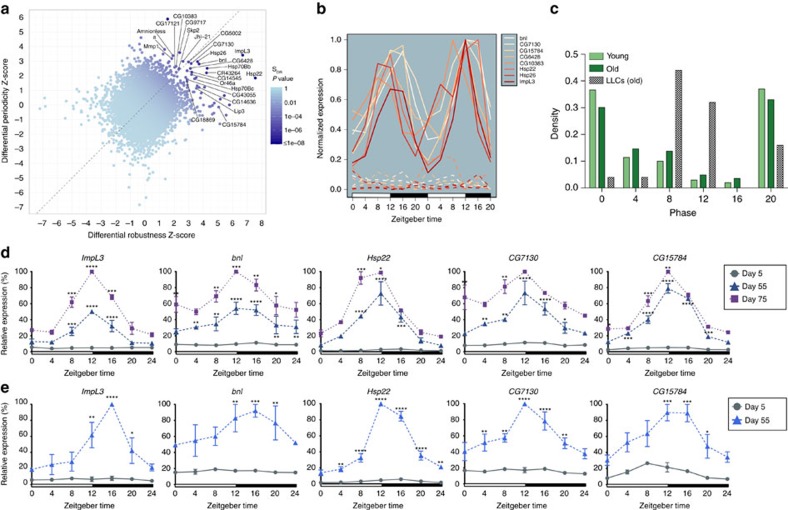
Identification of late-life cyclers (LLCs). (**a**) Differential periodicity Z-score is plotted against differential robustness *Z*-score (Methods); top LLCs from Supplementary Table 3 are labelled. Data points are coloured by differential rhythmicity score (*S*_DR_). (**b**) Superimposed min-max normalized RNA-seq profiles of known stress-responsive LLCs with peak expression >10 FPKM. Broken lines=day 5 data; solid lines=day 55 data. Each period of ZT 0–20 represents a distinct biological replicate. (**c**) Bars heights represent normalized counts of LLCs and genes rhythmic in young or old, which peak at indicated 4-h intervals (Methods). (**d**) qRT-PCR expression of LLCs in heads of females at age 5, 55 and 75 days. (**e**) qRT-PCR expression of LLCs in heads of young and old males. (**d**,**e**) Data are mean (from two cycles)±s.e.m. Expression is per cent of peak expression in the oldest group. *P≤0.05; **P≤0.01; ***P≤0.001; ****P≤0.0001 (two-way ANOVA with Bonferroni correction; day 55 change is relative to day 5 values, and day 75 change is relative to day 55 values).

The most unexpected outcome of our analysis was the identification of a number of genes that adopted *de novo* rhythmic expression or robustly increased amplitudes in old flies. We named this gene subset ‘late-life cyclers' or LLCs. To computationally identify LLCs, we developed a scoring metric called the differential rhythmicity score (*S*_DR_). The *S*_DR_ accounts equally for both the difference in the rhythmicity score, defined as the negative log of the ARSER *P* values for the respective age groups, and the differential robustness, or the log fold change in the max–min expression (see Methods). We identified genes with significant *S*_DR_ values at an FDR of 0.05 (Fig. 3a, Supplementary Fig. 4a, Supplementary Table 3)^23^. This method successfully identified genes with strong loss or gain in expression rhythmicity with age; however, some LLC-like genes of potential interest that do not make the cutoff are shown in Supplementary Fig. 4b. Another set of genes with rhythms abolished during aging are listed in Supplementary Fig. 4c. A complete list of genes ranked by *S*_DR_ can be found in Supplementary Data 2.

Supplementary Table 4 summarizes properties of the top 25 LLCs. 21 have predicted orthologs in humans according to the DRSC Integrative Ortholog Prediction Tool (DIOPT (ref. 24)). Functional analysis using DAVID v6.7 (ref. 25) revealed the terms ‘heat shock' and ‘stress response' to be enriched among the top LLCs (FDR 0.05). Published microarray data sets reported 16 of the 25 LLCs to be upregulated under oxidative stress (OS) when tested at one unspecified time of day^26^,^27^,^28^,^29^ (Supplementary Table 4). Figure 3b shows superimposed RNA-seq expression plots for LLCs that were upregulated by OS in at least two previous single-time-point studies. Strikingly, all of these genes peaked within two hours of night onset, suggesting that they may be governed by common regulatory mechanisms. The strong enrichment of LLCs peaking in the late day/early night starkly contrasts with the global phases of genes rhythmic in young or old flies, which peaked predominantly in the early day/late night (Fig. 3c).

We focused on the five LLCs with the most dramatic gains of rhythmicity for further investigations: small heat shock protein *Hsp22*; fibroblast growth factor ortholog *bnl*; lactate dehydrogenase (*ImpL3*); Hsp40-like *CG7130*; and *CG15784*, which bears homology to the mammalian histidine-rich glycoprotein (*HRG*) according to DIOPT. Independent qPCR experiments in flies aged to 5, 35, 55 and 75 days confirmed increased expression of these genes in 55-day old and showed even further increase in heads of very old (75 days) females (Fig. 3d). Middle-aged females already showed mild increase in the expression of these genes (Supplementary Fig. 5). Altogether, these data reveal LLC mRNA levels exhibit strong age-dependence. These effects are not sex dependent as LLCs showed similar expression changes in heads of 5-day versus 55-day males (Fig. 3e).

### Oxidative stress induces LLC rhythms in young flies

**Figure 4:**
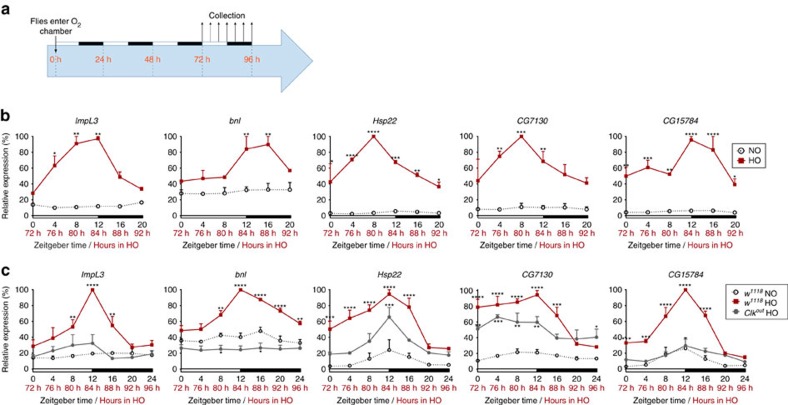
Oxidative stress-induced LLC rhythms in young flies. Heads of young females were collected in 12:12 light/dark (LD) at 4-h intervals on the 4th day in hyperoxia (HO) or normoxia (NO) according to the experiment scheme in **a**. (**b**,**c**) qRT-PCR results for RNA from heads of flies in HO or NO. Data (mean±s.e.m.) are reported as per cent of peak expression in LD under HO. *n*≥2 biological replicates. *P≤0.05; **P≤0.01; ***P≤0.001; ****P≤0.0001 (data from each genotype in HO are compared by two-way ANOVA with Bonferroni correction relative to *w* flies in NO). (**b**) *w* flies in NO and HO. (**c**) *w* flies in HO and NO; *Clk*^*out*^ flies in HO.

We hypothesized that oxidative stress (OS), which increases during aging^14^,^30^, might play a role in the rhythmic activation of LLCs in old flies. To test this, we exposed young *w* flies to continuous hyperoxia (HO; 100% O_2_). As the maximum lifespan under these conditions was 5–6 days, we collected flies at 4 h intervals on the fourth day after HO onset (Methods, Fig. 4a). Remarkably, HO induced robust rhythmic expression of the tested LLCs in these young flies (Fig. 4b), similar to those seen in old flies (Fig. 3d). Furthermore, HO reproduced in young flies the opposing changes in *per* and *tim* expression observed during aging (Fig. 2b, Supplementary Fig. 6a). These results suggest that oxidative stress contributes to rhythmic LLC upregulation in aging flies.

Because constant hyperoxia induced rhythmic rather than constitutive LLC transcription, we tested whether CLK is involved in LLC regulation by measuring their expression in young *Clk*^*out*^ mutants collected on the 4th day in HO. Remarkably, this exogenous OS failed to significantly upregulate *ImpL3*, *bnl* and *CG15784* in *Clk*^*out*^ flies relative to *w* controls in normoxia (Fig. 4c). Even for heat-shock LLCs *Hsp22* and *CG7130*, the upregulation in HO-treated *Clk*^*out*^ flies was less significant than in HO-treated *w* flies. These results support an essential role for CLK in promoting rhythmic LLC upregulation during OS.

Interestingly, although *tim* expression in *Clk*^*out*^ mutants was arrhythmic as expected, *per* expression in these HO-treated mutants remained weakly rhythmic, similar to several LLCs (Supplementary Fig. 6b). This suggests that CLK-independent mechanisms also contribute to rhythmic upregulation of *per* and some LLCs during OS.

### Age-induced expression of putative primary piRNAs

**Figure 5:**
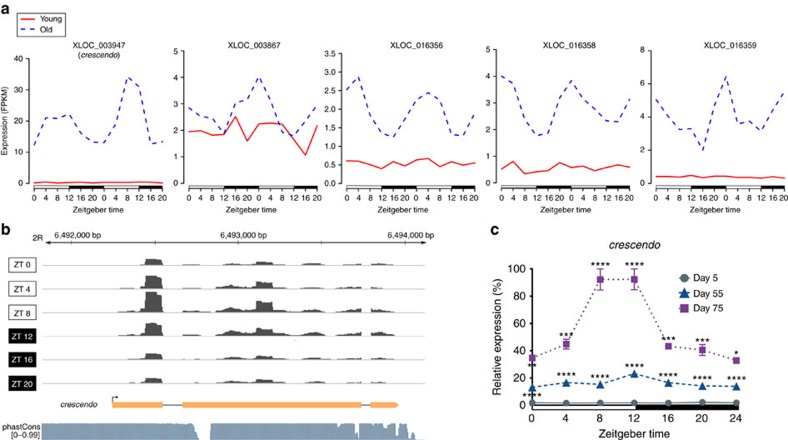
Age-induced circadian expression of novel genes. (**a**) RNA-seq expression plots for the five LLC-like novel genes; each period of ZT 0–20 represents a distinct biological replicate. XLOC IDs were assigned by Cuffmerge. (**b**) Browser image of the *crescendo* transcript model and corresponding RNA-seq reads from merged replicate datasets of old flies, with the same *y*-axis range across time points. The dm6 phastCons conservation tracks were downloaded from the UCSC Genome Browser website. (**c**) *crescendo* expression in heads of females on day 5, 55 and 75 by qRT-PCR is reported as per cent of peak level at day 75. Data are mean (from two cycles)±s.e.m. *P≤0.05; **P≤0.01; ***P≤0.001; ****P≤0.0001 (two-way ANOVA with Bonferroni correction; day 55 change is relative to day 5 values, and day 75 change is relative to day 55 values).

We performed *de novo* transcript assembly using StringTie^31^ and Cuffmerge^19^ and found 154 unannotated genes with multi-exonic transcripts (Supplementary Data 3). Twenty two of these were rhythmic in old flies (Supplementary Table 4), and five exhibited LLC-like behaviour (Fig. 5a). Among these five, only one (hereafter ‘*crescendo*') is conserved across several insects according to the 27 insect alignment and associated phastCons analysis from UCSC Genome Bioinformatics^32^ (Fig. 5b). While *crescendo* partially overlapped both a TE and a piRNA cluster annotation in the sense strand, the other four fully overlapped transposable elements (TEs) in antisense and mature Piwi-interacting RNAs (piRNAs) in the sense strand^33^, implicating these four as primary piRNA transcripts^34^ (Supplementary Fig. 7).

We measured *crescendo* expression by qPCR in heads of 5-, 55- and 75-day old females, and observed an exponential increase with age, with the greatest increase occurring between days 55 and 75 (Fig. 5c). As it oscillated in the same phase as other tested LLCs, we measured *crescendo* levels in HO-exposed flies; however, it was not significantly upregulated (Supplementary Fig. 8), suggesting that *crescendo* is not induced directly by HO but may be stimulated in response to other age-associated changes.

### Differential gene expression independent of time-of-day

**Figure 6:**
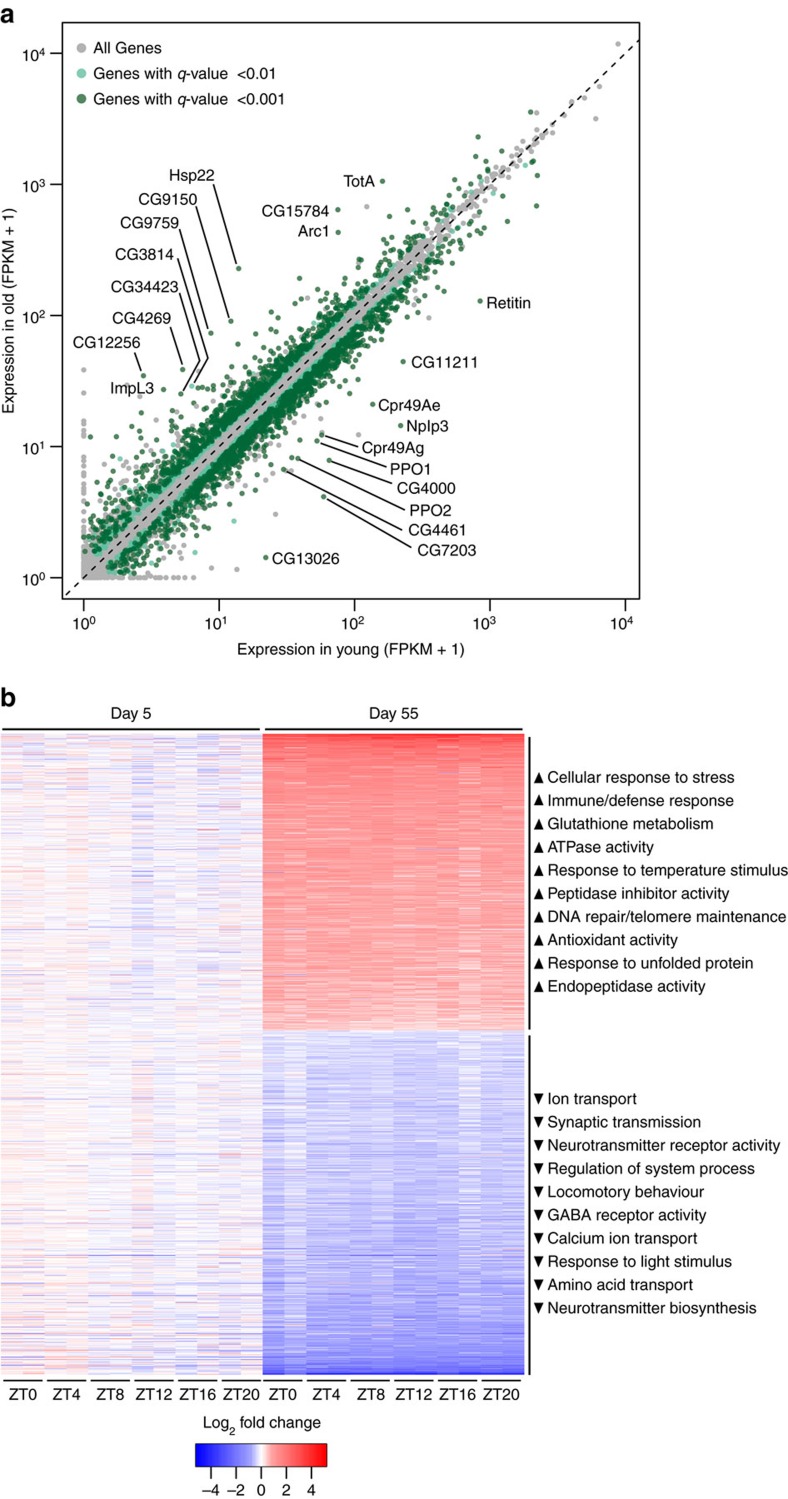
Age-related and time-averaged differential expression. (**a**) Scatterplot showing average daily gene expression in heads of old versus young flies according to RNA-seq. Data represent mean across six time points each with two biological replicates; differentially expressed genes are coloured according to *q*-values reported by Cuffdiff. Genes with significant (FDR 0.05) disparities in average daily expression between two biological replicates were included but not coloured. Labelled genes have an FPKM≥20 in young or old flies as well as an old/young fold change ≥5. (**b**) Heat map showing gene expression changes during aging according to RNA-seq. Each row represents a differentially expressed gene (FDR 0.01) with fold change ≥1.5 and a minimum FPKM of 1 in either young or old. Genes with significant (FDR 0.05) disparities in average daily expression between two biological replicates were excluded. For each sample (column), colour corresponds to fold change given by dividing the sample's FPKM by the average FPKM in young. Data from biological replicates are grouped in adjacent columns. Right: terms representing the top ten clusters of GO terms reported as enriched among up- or down-regulated genes by DAVID.

Our round-the-clock data afforded a high-confidence measure of age-dependent changes in average expression level for all genes, by treating individual samples from different time points as replicates. Among genes with an FPKM>1 in young or old flies, we found 1,504 genes to be significantly downregulated by the age effect (FDR 0.01) and 1,307 genes to be upregulated (Fig. 6a; Supplementary Data 4). Of these, the 583 genes upregulated and the 676 genes downregulated by >50% during aging are shown in Fig. 6b. We found the ‘housekeeping gene' *Act5C* (actin) in the strongly upregulated subset (Supplementary Fig. 9), indicating that it is a poor endogenous control for age-dependent qPCR experiments in *Drosophila*. Notably, 33.5% of differentially expressed genes (FDR 0.01, fold-change ≥1.5) were robustly rhythmic in young flies, old flies, or both.

Functional analysis with DAVID revealed several enriched annotation clusters for differentially expressed genes. Gene ontology terms related to immune response, glutathione metabolism and response to cellular and genotoxic stress were enriched among upregulated genes; terms associated with neural function, locomotory behaviour, ion homoeostasis and response to entrainment cues were enriched among downregulated genes (Fig. 6; Supplementary Data 5).

## Discussion

This genome-wide study uncovers the diverse changes in daily RNA expression patterns that occur in heads of aging flies and provides new insights into mechanisms linking clock function and protection from oxidative damage. We show that during aging, several LLCs adopt *de novo* transcriptional rhythms that can also be induced in young flies by exogenous oxidative stress. Because OS directly promotes neurodegeneration in flies^26^ and mice^16^, LLCs may be a missing link underlying observations that age-related increases in OS are exacerbated by disruption of circadian clocks^8^,^14^, and that clock mutations accelerate OS-induced neurodegeneration in aging flies^15^ and mice^16^. Interestingly, a recent postmortem study of gene expression in the human brain reported some daily transcript levels to show significantly better correlations with sinusoidal curves in the ≥60-yr-old group than in the <40-yr-old group^35^, suggesting potential conservation of the LLC phenomenon.

In addition to identifying numerous annotated genes with *de novo* rhythmicity in old flies, we also identified LLC-like putative primary piRNAs overlapping transposons in antisense. Because transposon mobilization increases during aging and may contribute to age-related neuronal decline in *Drosophila*^36^, we propose that late-life activation of circadian piRNA expression is a novel strategy by which the molecular oscillator preserves genomic integrity during aging.

Our study provides first insights into the mechanism of LLC regulation, which involves CLK, the rate-limiting master regulator of circadian transcription in *Drosophila*^37^. Our data support a model in which the circadian system enlists LLCs late in life to mitigate damage resulting from potentially diverse sources of cellular and genotoxic stress that accumulate during aging.

## Methods

### Fly rearing and hyperoxia treatment

*Drosophila melanogaster* were raised on a standard yeast (35 g l^−1^), cornmeal (50 g l^−1^) and molasses (5%) diet at 25±1 °C, under a light-dark (LD) 12:12h regimen. Mated flies were kept in groups of 50 males or 50 females in 300 ml round bottom polypropylene ventilated bottles (Genesee Scientific, San Diego, CA). Diet was changed three times a week without anaesthesia. The following genotypes were used in this study: *w*^*1118*^ (control) and *Clk*^*out*^ (ref. 38). To induce oxidative stress by hyperoxia (HO), 5-day-old flies of each genotype were placed in clear, airtight chambers with 100% O_2_ flow-through at atmospheric pressure, starting at ZT0. Control flies remained in normoxia (NO) next to the chambers. Two biological replicates of 25–50 flies each in HO or NO were collected simultaneously every 4 h for one 24 h cycle starting at 72 h after HO onset in LD or DD as shown in Fig. 4a.

### RNA extraction and qRT- PCR

One biological replicate of 50 flies was collected every 4 h for two 24 h cycles starting at ZT0 (lights on) for qRT-PCR. Each sample of 25–50 fly heads was homogenized in TRIzol Reagent (Thermo Fisher, Waltham, MA) using a Kontes handheld motor and pestle, and RNA was extracted according to the manufacturer's instructions. Samples were treated with rDNase I (Takara, Japan), followed by a phenol/chloforom extraction and ethanol/sodium acetate precipitation. Complementary DNA (cDNA) was synthesized from 1 μg of RNA using the iScript cDNA synthesis kit (Bio-Rad, Hercules, CA), or the Maxima First Strand cDNA Synthesis Kit for RT-qPCR (Thermo Fisher, Waltham, MA). Real-time PCR was performed with Power SYBR Green PCR Master Mix (Thermo Fisher, Waltham, MA) on a StepOne Plus Real-Time machine (Applied Biosystems, Foster City, California). Primers were obtained from Integrated DNA Technology (Coralville, Iowa), and all primer sets were verified to have >90% efficiency. Primer sequences are given in Supplementary Table 6. Data were analysed using the 2^−ΔΔCT^ method, using *Decapping protein 2* (*DCP2*) as the endogenous control for normalization. *DCP2* was selected based on its low variance between time points and ages according to RNA-seq and qPCR (Supplementary Fig. 9). Data for ZT 24 of cycle 1 at day 5 is repeated for ZT 0 of cycle 2 at day 5 for the experiment in females aged to 5, 55 and 75 days.

### Western blotting

Heads of 5 and 55 day-old females (three biorepeats of 20 heads per timepoint for each age) were homogenized in Laemmli buffer, sonicated, boiled at 100 °C for 5 min and centrifuged at 12,000*g* at 4 °C. A constant ratio of the buffer (7 μl per head) was used to ensure equal protein loading and separation on NuPAGE 4–12% gradient acrylamide gel (Life Technologies). Proteins were transferred to the 0.45 μm polyvinylidene fluoride (PVDF) Immobilon-FL membrane (Millipore Billerica, MA) and stained for 5 min with REVERT Total Protein Stain Kit (Li-Cor Biosciences). After staining, membranes were scanned in the 800 channel on the Odyssey Infrared Imaging system to quantify the total protein. The staining was reversed using the same kit, and the membranes were incubated in 1 × TBST (10 mM Tris, 0.15 M NaCL, 0.1% Tween-20, pH 7.5)+5% dry milk for 2 h, then overnight at 4 °C with 1:15,000 anti-PER (gift from Dr J. Price)^39^ in blocking buffer. Membranes were treated for 2 h with 1:20,000 goat anti-rabbit IRDye800 (catalogue # 926–32211, LI-COR Biosciences, Lincoln, NE) diluted in Odyssey Blocking Buffer. After washes, PER signal was quantified relative to total protein using the LI-COR Odyssey Infrared Image Studio software according to the manufacturer's instructions. Uncropped version of the PER western blot is present in Supplementary Fig. 3.

### Fragment library preparation and RNA sequencing

Following poly(A) selection of RNA samples purified as above, strand-specific cDNA libraries were prepared using Illumina's TruSeq stranded mRNA kit (100 bp paired-end) following the manufacturer's directions, then sequenced on Illumina HiSeq 2000 with parallel samples from young and old heads multiplexed in the same lane.

### Read alignment and quantification of transcript abundance

Raw RNA-seq reads were filtered to exclude reads with mean quality scores <30 and to trim 3′ ends with mean quality <30, using the program skewer^40^. Filtered reads were aligned to the *Drosophila melanogaster* genome (BDGP release 6.06/dm6) using TopHat version 2.0.14 with a max intron length of 10,000. Aligned reads from individual time points were submitted to StringTie v1.2.0 for novel transcript assembly, with minimum junction coverage of 2; release 6.06 of the FlyBase genome annotation was used to guide the assembly process. The resulting list of novel transcripts was refined with Cuffmerge from the Cufflinks package (v2.2.1). Cuffdiff was used to compute abundance of genes and transcripts in units of Fragments Per Kilobase of transcript per Million mapped reads (FPKM) for novel genes and isoforms, separately from those annotated in the FlyBase genome annotation. We note that expression of some *de novo* assembled transcripts, including the four LLC-like putative primary piRNA transcripts (Fig. 5), could not be reliably quantified because most of their reads aligned with high per cent identity to multiple genomic loci according to Bowtie 2 (ref. 41). However, each of these four assembled transcripts aligned only once with 100% identity according to the UCSC Genome Bioinformatics BLAT tool.

Because of their short length resulting in unstable FPKM calculations, pre-microRNA hairpins were excluded from all subsequent analyses.

All genome browser tracks showing RNA-seq reads were generated in the Integrative Genomics Viewer (IGV)^42^.

### Gene expression rhythmicity detection

Rhythmic transcripts with 24-h periodicity were identified using ARSER (ref. 20), JTK_CYCLE^43^ and empirical JTK_CYCLE^44^ (Supplementary Table 1; Supplementary Fig. 2a). For all three programs, input data was formatted as a series of two daily cycles. The *P* value used as a significance threshold for empirical JTK_CYCLE was the empirical *P* value. Genes reported as rhythmic have a median expression ≥1 FPKM, a max/min fold-change ≥1.5 and a *P* value≤0.05 in accordance with published thresholds for rhythmicity detection^5^,^45^. Although all three methods showed substantial agreement (Supplementary Fig. 10), ARSER showed the strongest proportional overlap between genes rhythmic in old versus young flies (Supplementary Table 7). Thus, to be conservative when identifying age-dependent changes in rhythmicity, we used ARSER output for subsequent analyses. For basic comparisons, we define ‘highly rhythmic' as *P* value≤0.01, and arrhythmic as *P*>0.5. For comparison of overlap between our set of genes rhythmic in young females and genes reported by others as rhythmic in heads of young males^3^, we evaluated the per cent overlap with genes that mapped to IDs in Flybase release 6.06.

### Differential rhythmicity analysis

We assigned an *S*_DR_ to each gene having an ARSER *P* value<1 and median expression ≥1 FPKM in young or old flies, and nonzero expression in at least one time point in young and old flies. We then assigned *P* values to these normally distributed *S*_DR_ values and subsequently computed their false discovery rates (FDRs) using the BH procedure to adjust for multiple hypothesis testing (Supplementary Fig. 4a and b)^23^. Among the resulting set of significantly (FDR 0.05) differentially rhythmic genes, we defined our top LLCs as those rhythmic in old flies (max/min fold-change≥1.5 and ARSER *P* value≤0.05) with at least one isoform satisfying the rhythmicity criteria imposed at the gene level in old flies (Fig. 3a).

When identifying genes with the strongest improvements in rhythmicity with age, we found that many genes either showed trivial expression levels and low peak/trough fold change in young but robust amplitudes in old, or showed enhanced precision in the periodicity of their expression with age. A few, including *ImpL3*, fell into both of these categories. We sought to define a metric that would incorporate these two patterns of differential rhythmicity, and to this end we developed a differential rhythmicity score calculated as follows:


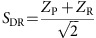


*S*_DR_ is the sum of two Z-scores divided by 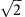; the factor of 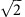 in the denominator ensures that it obeys a standard normal distribution. The first term, *Z*_P_, is a Z-score computed for the age-dependent change in periodicity, 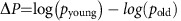, using the *P* values from ARSER in young and old. The second term *Z*_R_, is a Z-score for the differential robustness Δ*R*, the log fold change in the effective amplitude, given by


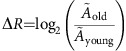


where 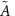 is the effective amplitude, max FPKM–min FPKM. Each of these Z-scores showed a normal distribution, as did the combined score *S*_DR_ (Supplementary Fig. 4a). A *P* value was computed for each *S*_DR_ using a Gaussian distribution based on the fit to the empirical distribution of *S*_DR_ values, and the BH procedure was used to compute FDRs.

The custom Perl scripts that we used to implement this analysis can be found at http://hendrixlab.cgrb.oregonstate.edu/LLCs.html

For the bar plots in Fig. 3c, phases from ARSER were rounded to the nearest integer and mapped to bins of size (4*n*−2, 4*n*+2) for *n*=0, 1, ..., 5, where each integer 4*n* is a time point sampled for RNA-seq.

### Differential expression analysis

To identify age-induced differential expression independent of time of day, aligned reads for each individual data point (single fly cohort at single-time-point) were treated as replicates segregated by age into either the day 5 or day 55 group. Cuffdiff was run to compute average expression over samples for a given age, and to identify significantly differentially expressed genes. Using all the samples for a given age in a batch analysis using Cuffdiff properly identifies genes that are consistently differentially expressed due to age, regardless of time of day. The DAVID Functional Annotation Tool (https://david.ncifcrf.gov/summary.jsp) was used to identify enriched functional annotations among significantly upregulated or downregulated genes having a minimum FPKM of 1 in young or old and a fold change ≥1.5. In addition, 525 out of the 582 upregulated genes and 572 of the 676 downregulated genes mapped to DAVID IDs. For annotation we included biological process and molecular function gene ontology (GO) terms, as well as KEGG pathways. For functional annotation clustering we used default classification stringency (Medium). The top 10 annotation clusters are summarized next to the heat map in Fig. 6a. Because DAVID does not provide names for the annotation clusters, we assigned names that globally represented the terms present in each cluster. Complete results of GO analyses are presented in Supplementary Data 5.

### Data availability

Data for RNA-seq and processed files have been deposited to NCBI Gene Expression Omnibus (GEO) under the accession number GSE81100. RNA-seq expression plots for all FlyBase genes and isoforms are available at: http://hendrixlab.cgrb.oregonstate.edu/youngAndOldExpression.html.

All other data supporting the findings of this study are included in the manuscript and its supplementary files or are available from the corresponding authors on request.

## Additional information

**How to cite this article:** Kuintzle, R. C. *et al*. Circadian deep sequencing reveals stress-response genes that adopt robust rhythmic expression during aging. *Nat. Commun.* **8,** 14529 doi: 10.1038/ncomms14529 (2017).

**Publisher's note**: Springer Nature remains neutral with regard to jurisdictional claims in published maps and institutional affiliations.

## Supplementary Material

Supplementary Information Supplementary Figures, Supplementary Tables and Supplementary References

Supplementary Data 1 Rhythmicity Classification for Genes and Transcripts. Sheet one contains ARSER-reported p-values, q-values (BH-corrected p-values), and phases for all genes, as well as median expression values and max/min expression fold change (FC), computed after adding a pseudo-count (lowest nonzero expression value) to the max and min values to avoid divide by zero errors. Sheet 2 contains p-values and BH q-values for three oscillatory clock genes according to ARSER, JTK_CYCLE, and empirical JTK_CYCLE (Methods).

Supplementary Data 2 LLC Analysis and Results. Differential rhythmicity scores (SDR) and statistics for all genes with an ARSER p-value < 1 in young or old flies, median expression ≥ 1 FPKM in young or old, and nonzero expression in at least one time point in young and old flies to prevent divide-by-zero errors. ZP = Z-score for the periodicity difference; ZR = Z-score for the robustness difference; Max-Min = difference in highest and lowest expression values. The Z-score column corresponds to the SDR Z-score computed using mean and standard deviation values from a Guassian fit. For more details, see Methods.

Supplementary Data 3 Putative Novel Gene Annotation. This file in gtf format contains predicted genomic locations for all multi-exonic genes from novel transcript assembly (Methods) that were not associated with genes annotated in release 6.06 of the FlyBase gene model annotation.

Supplementary Data 4 Age-Related Differential Expression. Raw Cuffdiff output for daily averages of old flies vs. young (Methods).

Supplementary Data 5 DAVID Functional Annotation Output. Genes strongly up- or down-regulated (FDR 0.01) according to Cuffdiff, with an age-related fold change ≥ 1.5 and an FPKM ≥ 1 in either young or old, were analyzed for functional annotation enrichment using DAVID Functional Annotation Tool (https://david.ncifcrf.gov/summary.jsp). Genes with significant (FDR 0.05) disparities in expression between biological replicates according to Cuffdiff were excluded. This file contains DAVID Functional Annotation Chart and Functional Annotation Clustering output. See Methods for further information.
