# Supplementary material for: Circadian deep sequencing reveals stress-response genes that adopt robust rhythmic expression during aging
Source: Nat Commun. 2017 Feb 21;8:14529. doi: 10.1038/ncomms14529 (PMC5321795; doi:10.1038/ncomms14529)
Supplement: Supplementary Information — Supplementary Figures, Supplementary Tables and Supplementary References [file ncomms14529-s1.pdf]

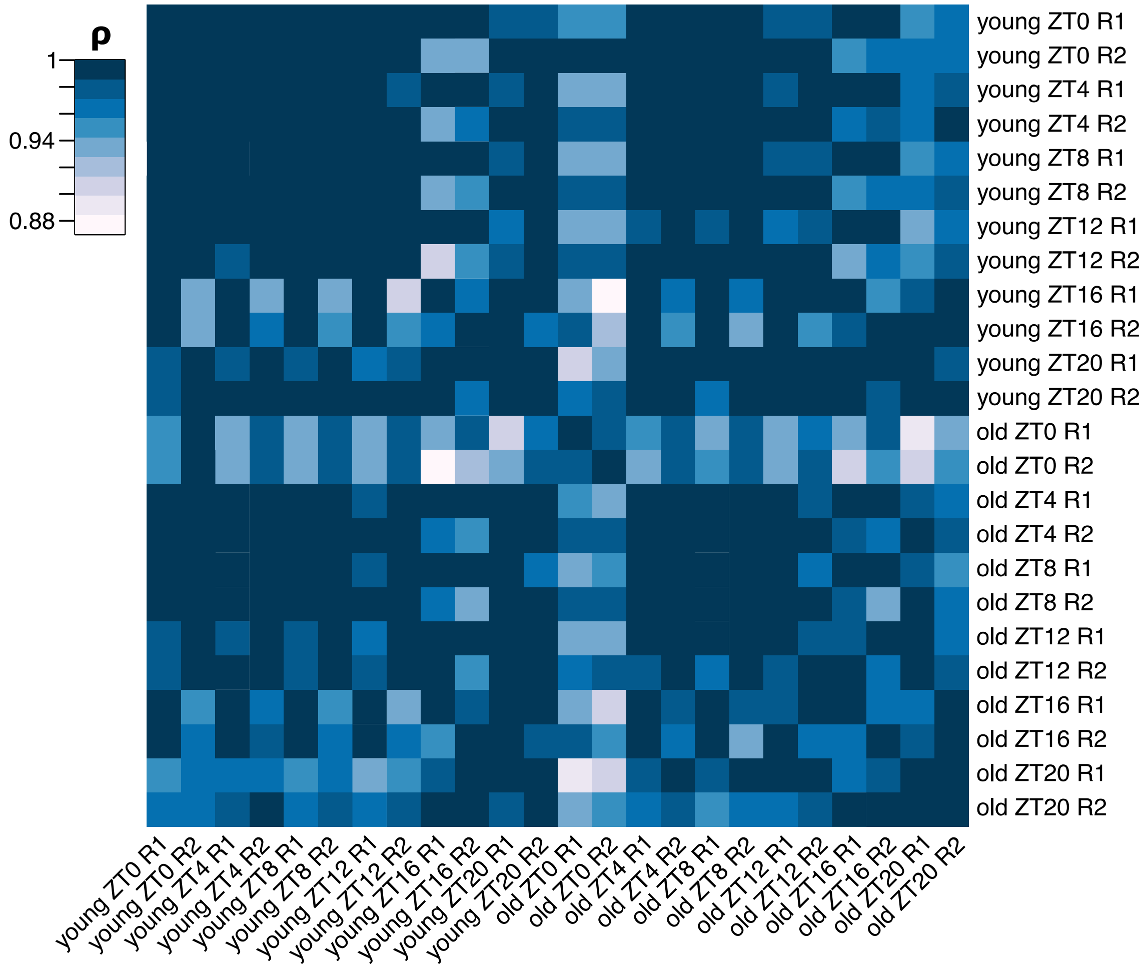

Supplementary Figure 1. Biological Replicates Show Strong RNA-seq Gene Expression Correlations.

Heat map showing gene expression Pearson's correlations for all RNA-seq data sets, calculated with custom Perl scripts using the module Statistics::Regression from Cuffdiff-computed gene abundances.

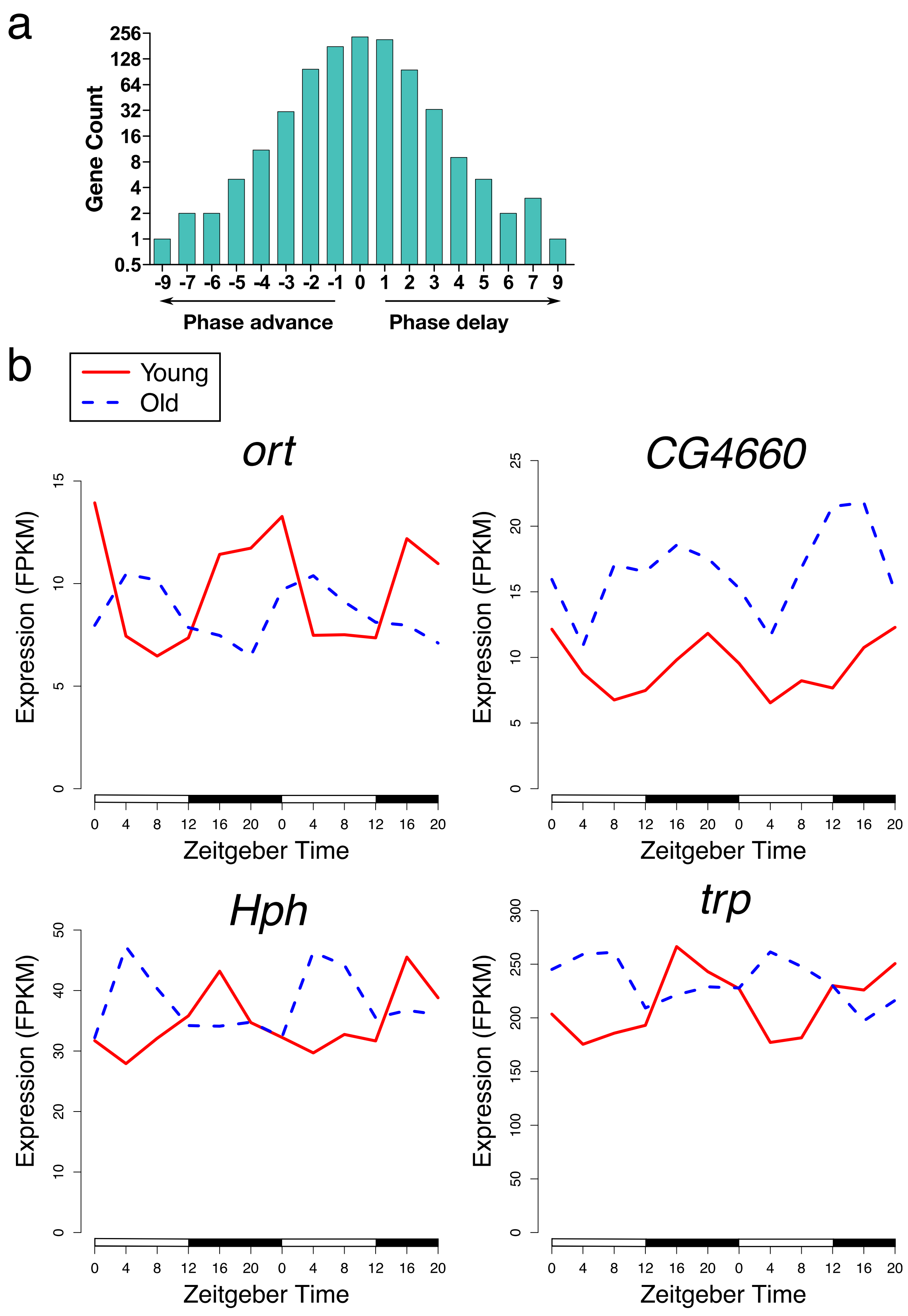

Supplementary Figure 2. Age-induced Phase Shifts.

(**a**) Bar plot showing phase advances and phase delays among genes rhythmic in both young and old at $p \leq 0.05$ according to ARSER; these genes were also required to have a median FPKM ≥ 1 and a max/min expression fold change ≥ 1.5 in young and old. ARSER-reported phases were rounded to the nearest integer; phases rounded to 24 were labeled as 0. (**b**) Examples of RNA-seq expression plots for rhythmic genes with phase shifts ≥ 4 hours. The bottom two genes show significantly rhythmic expression but have max/min fold changes < 1.5. The products of these genes according to FlyBase are as follows: *CG4660*, putative thioesterase; *ort,* GABAA receptor; *Hph,* dioxygenase enzyme involved in response to hypoxia and DNA damage; and *trp,* light-activated calcium ion channel. Each period of Zeitgeber Time (ZT) 0-20 represents a distinct replicate.

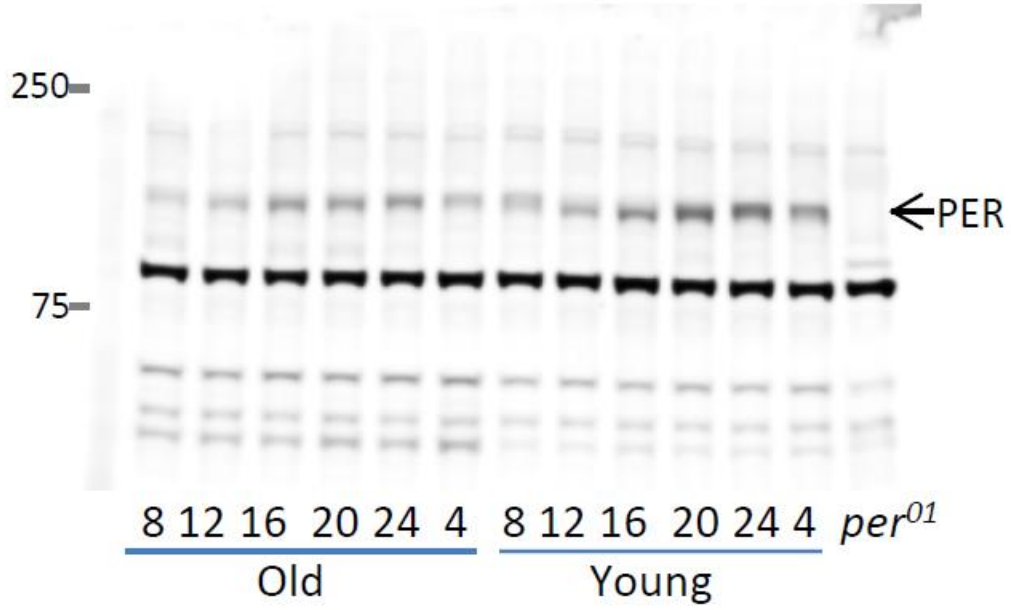

Supplementary Figure 3. PER Western Blot.

Entire PER Western Blot. PER protein is marked by the arrow. Approximate position of markers is indicated. Original markers were pre-stained with the REVERT Total Protein Stain Kit (see Methods) and are not visible through the 800 channel on the Odyssey Infrared Imaging system.

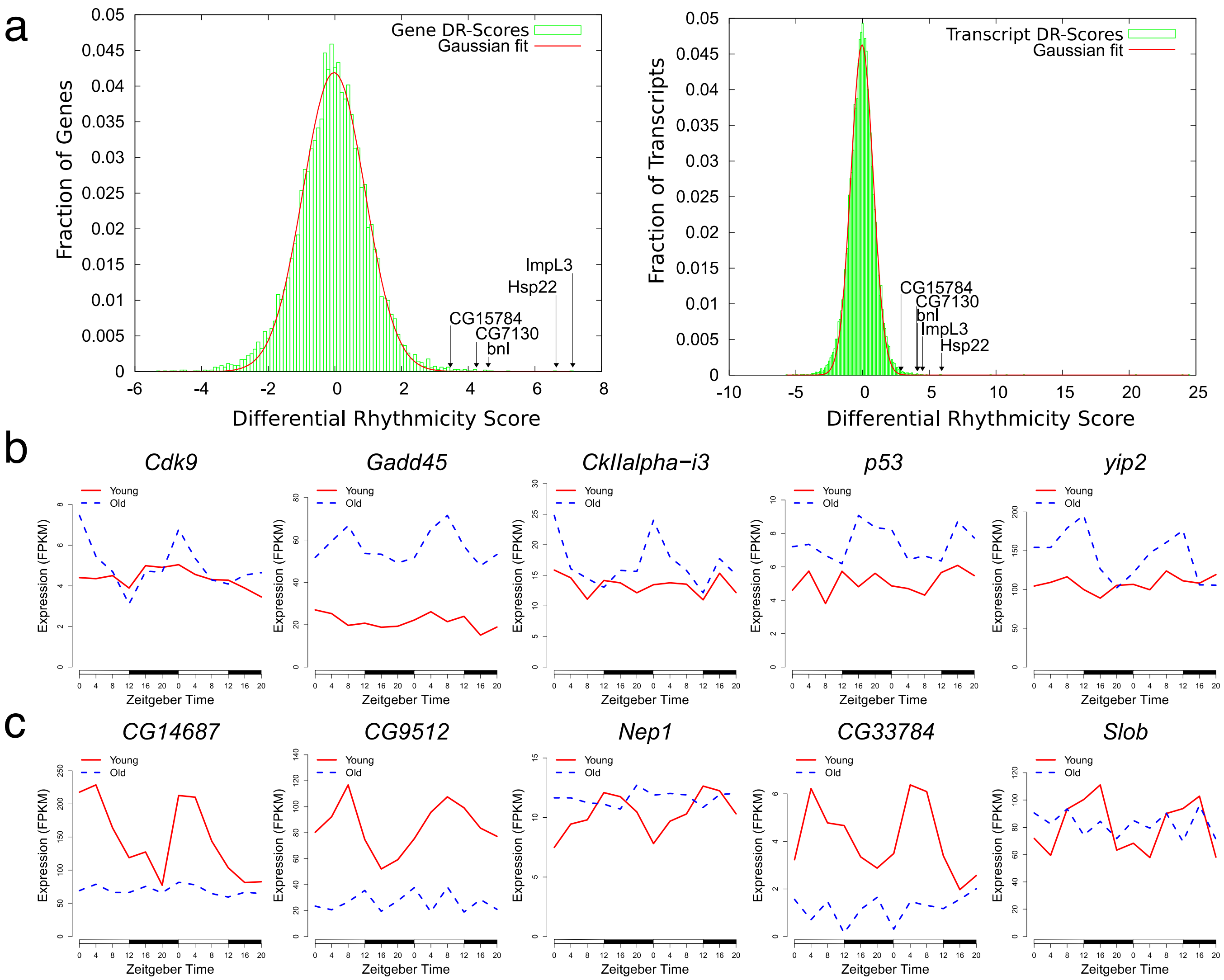

Supplementary Figure 4. Late-Life Cycler Analysis and Examples.

(**a**) Histograms of differential rhythmicity scores (S_{DR}) are shown for genes (left) and transcripts (right). A bin size of 0.1 was used in creating both histograms. Gaussian fit equations were computed using Gnuplot's "fit" function. (**b**-**c**) RNA-seq expression plots. Each period of ZT0-20 represents a distinct replicate. (**b**) LLC-like genes that did not make the FDR cutoff include, but are not limited to: cyclin-dependent kinase 9 (*Cdk9*), a component of the positive transcription elongation factor P-TEFb; growth arrest and DNA damage-inducible protein (*Gadd45*); CKII-α subunit interactor-3 (*CkIIalpha-i3*); transcription factor *p53*; and thiolase *yip2*. (**c**) Examples of early life cyclers (ELCs) include *CG14687*, oxidoreductase *CG9512*, Neprilysin 1 (*Nep1*), *CG33784*, and protein kinase *Slob*.

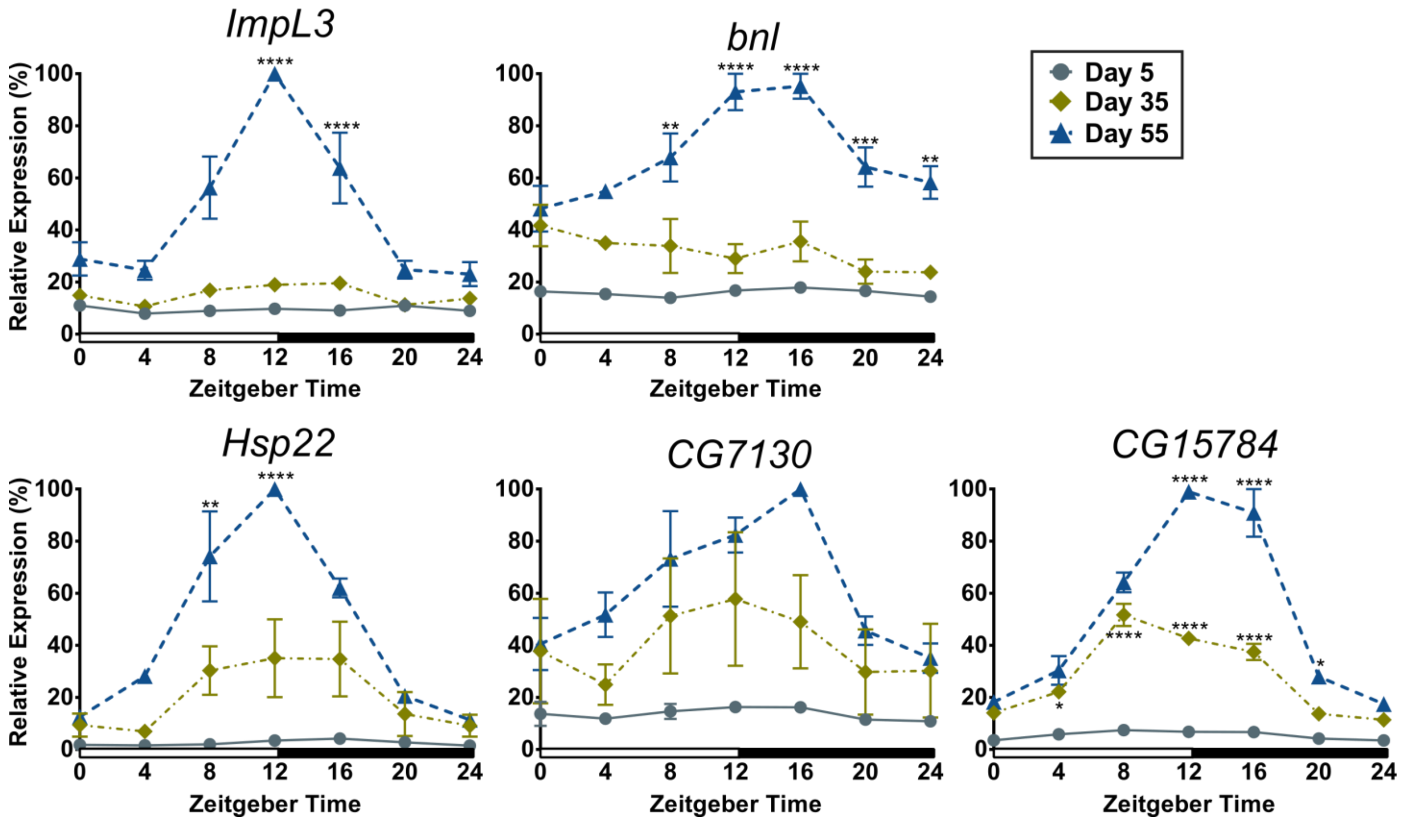

Supplementary Figure 5. Late Life Cycler (LLC) Expression at Young, Middle, and Old Age. qRT-PCR expression of LLCs in heads of *w* females at age 5, 35, and 55 days. Data are mean (from two cycles) ± SEM. Expression is percent of peak expression in the oldest group. *P≤.05; **P≤.01; ***P≤.001; ****P≤.0001 (two-way ANOVA with Bonferonni correction; day 35 change is relative to day 5 values, and day 55 change is relative to day 35 values).

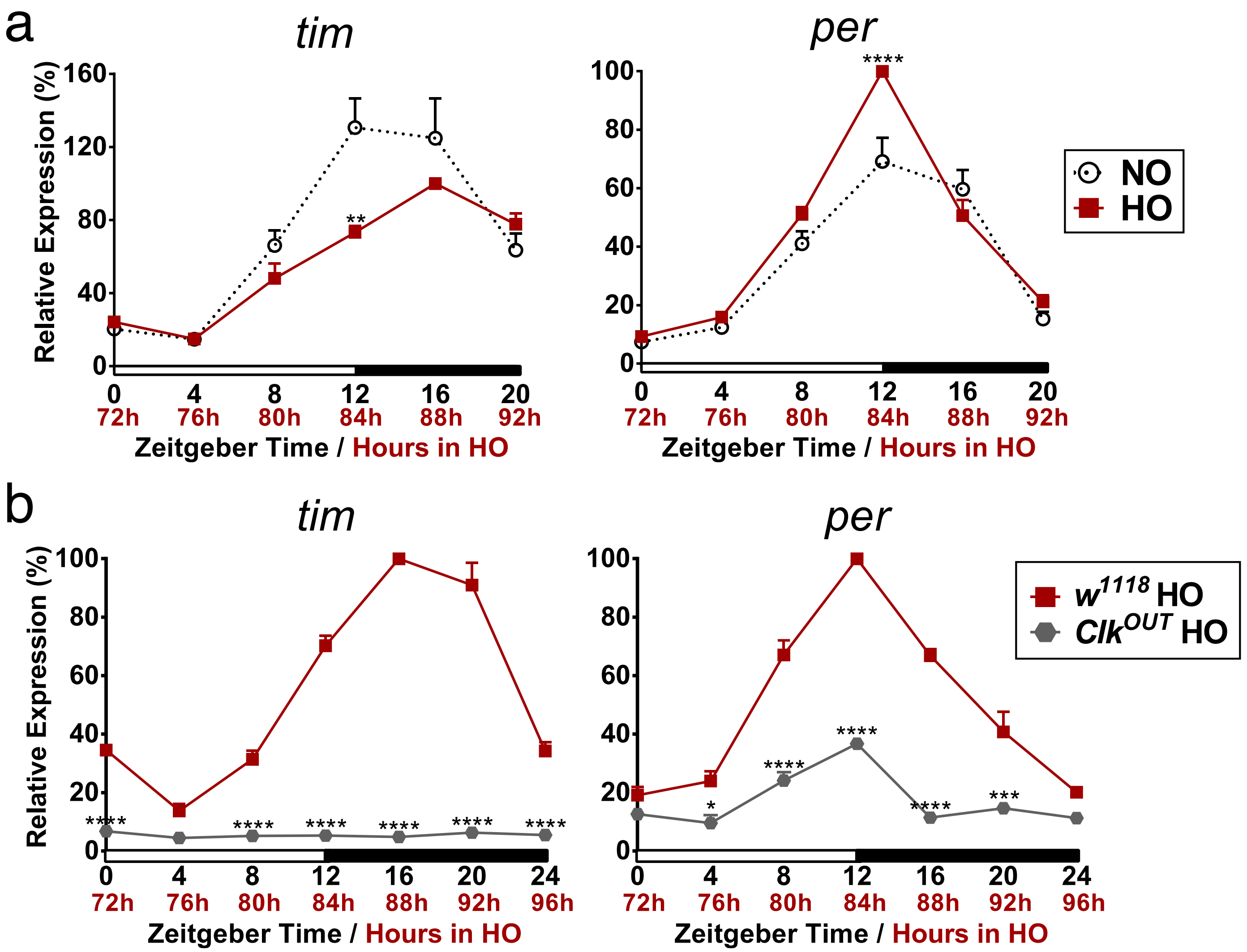

Supplementary Figure 6. Expression of *per* and *tim* in Hyperoxia.

(**a**-**b**) qRT-PCR results for RNA from heads of flies in hyperoxia (HO) or normoxia (NO). Data (mean ± SEM) are reported as percent of peak expression in 12:12 light/dark (LD) under HO; $n \geq 2$ biological replicates. *$P \leq .05$; **$P \leq .01$; ***$P \leq .001$; ****$P \leq .0001$. (**a**) *w* flies in NO and HO; data from HO flies are compared by two-way ANOVA with Bonferroni correction to data from NO flies. (**b**) *w* and Clk^{out} flies in HO; data in Clk^{out} are compared by two-way ANOVA with Bonferroni correction to data in *w* flies. For *per*, within individual genotypes, significant differences in expression between each time point from the trough as tested by one-way ANOVA are as follows: w^{1118}: ZT8 $p < 0.001$, ZT12 $p < 0.0001$, ZT16 $p < 0.001$, ZT20 $p < 0.05$; Clk^{out}: ZT8 $p < 0.01$, ZT12 $p < 0.0001$.

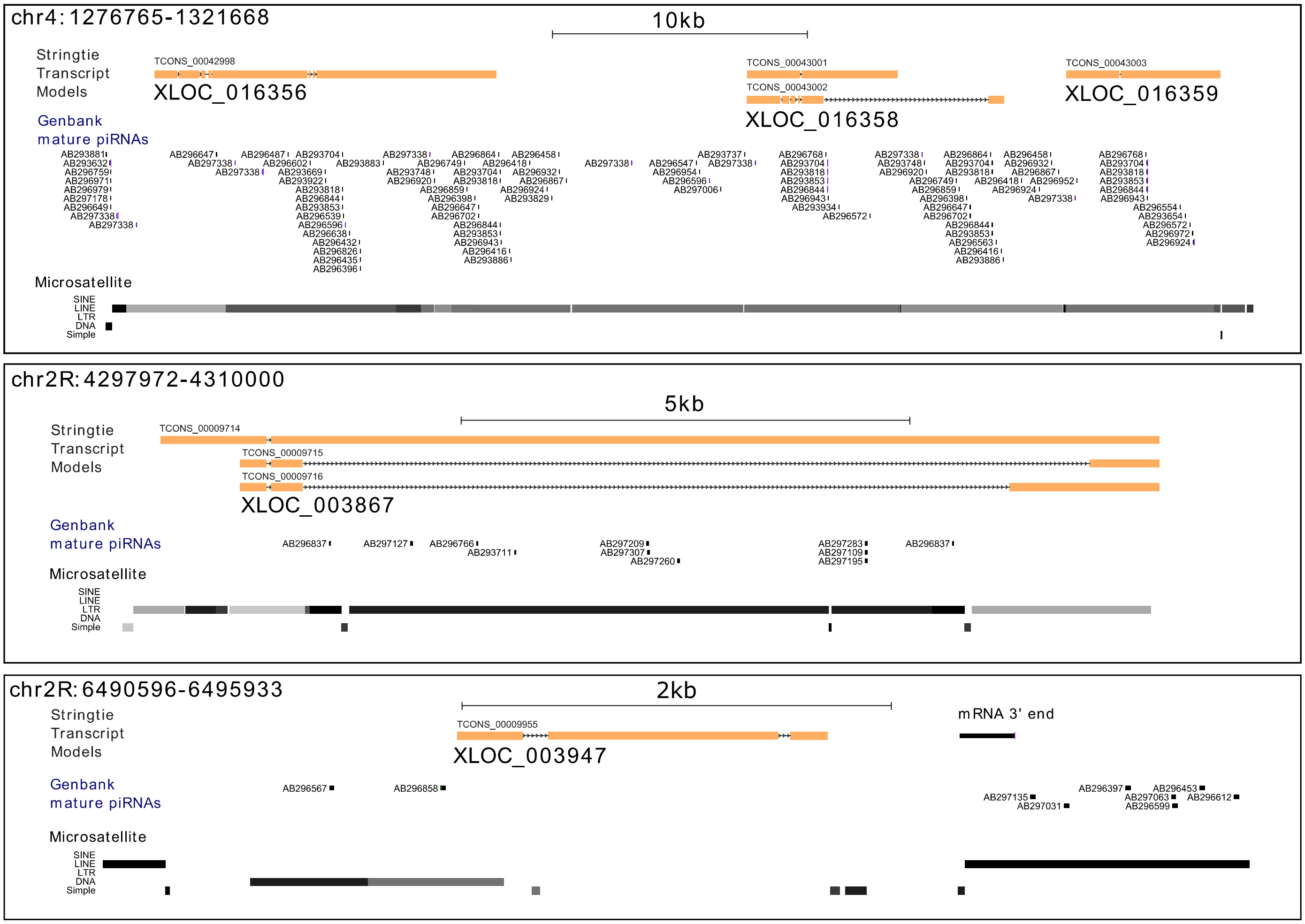

Supplementary Figure 7. Genome Browser View for Primary piRNA LLCs.

Stringtie transcript models in some cases consist of multiple isoforms (TCONS-IDs), for a given gene model (XLOC-IDs). All three gene models on chromosome 4 (chr4), as well as XLOC_003867 on chr2R, contain mature piRNA annotations in the sense strand relative to the piRNA transcript, and fully overlap transposable element (TE) annotations in antisense. The fifth gene model, XLOC_003947 (*crescendo*), is located near the end of piRNA cluster 42AB according to FlyBase (not shown), and partially overlaps a PROTOP (family P) DNA transposon in the same strand. Mature piRNA annotations for each locus are from Genbank, and Microsatellite tracks are from RepeatMasker. Both tracks were downloaded from the UCSC genome browser.

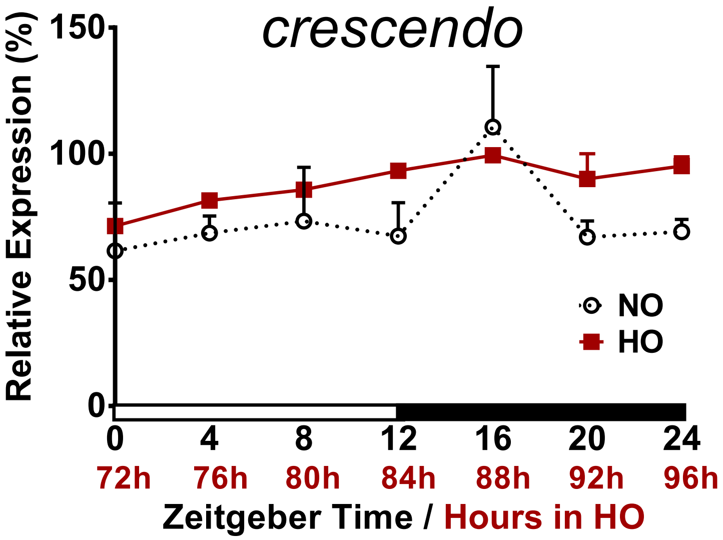

Supplementary Figure 8. Expression of the Novel Gene *crescendo* in Hyperoxia versus Normoxia. Expression of *crescendo* in heads of young *w* females collected at 4-hour intervals on the 4th day in hyperoxia (HO) or normoxia (NO). Data (mean ± SEM) are reported as percent of peak expression in NO. $n \geq 2$ biological replicates. No significant difference is seen between NO and HO at each timepoint as compared by two-way ANOVA with Bonferroni correction.

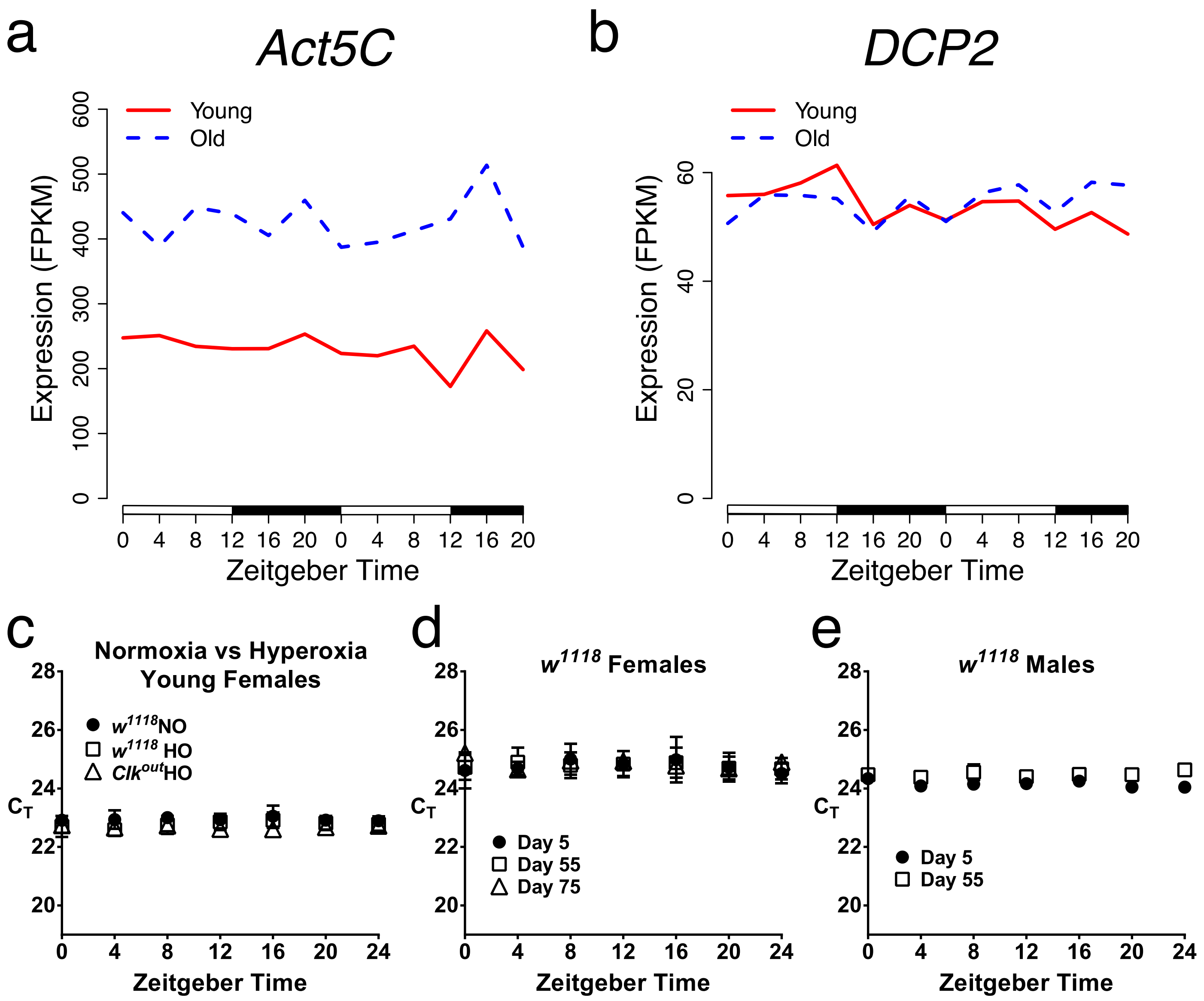

Supplementary Figure 9. Endogenous Control for qPCR.

RNA-Seq expression plots. Each period of ZT 0-20 represents a distinct replicate.

(**a**) *Act5C* (actin), a gene commonly used as an endogenous control for qPCR analyses in *Drosophila*, showed strong constitutive upregulation with age in female flies. (**b**-**e**) *DCP2* (Decapping protein 2) was selected for use as endogenous control in this study based on its low variance according to RNA-seq (**b**), subsequently confirmed by low variance in C_T in independent samples by qPCR (**c**-**e**). (**c**-**e**) Representative C_T values for *DCP2*, the reference gene used for all qRT-PCR performed. (**c**) Values from heads of young *w* females in Hyperoxia (HO) and Normoxia (NO), and young *Clk*^{out} females in HO; cDNA was

synthesized as described in Methods with the Thermo Scientific Maxima First Strand cDNA Synthesis kit. Data are mean ± SEM from two biological replicates collected over one daily cycle.

(**d**-**e**) Data are mean ± SEM from two daily cycles. cDNA was synthesized as described in Methods with the BioRad iScript cDNA synthesis kit using RNA from heads of females at age 5, 55, and 75 days (**d**) and from heads of males at age 5 and 55 days (**e**).

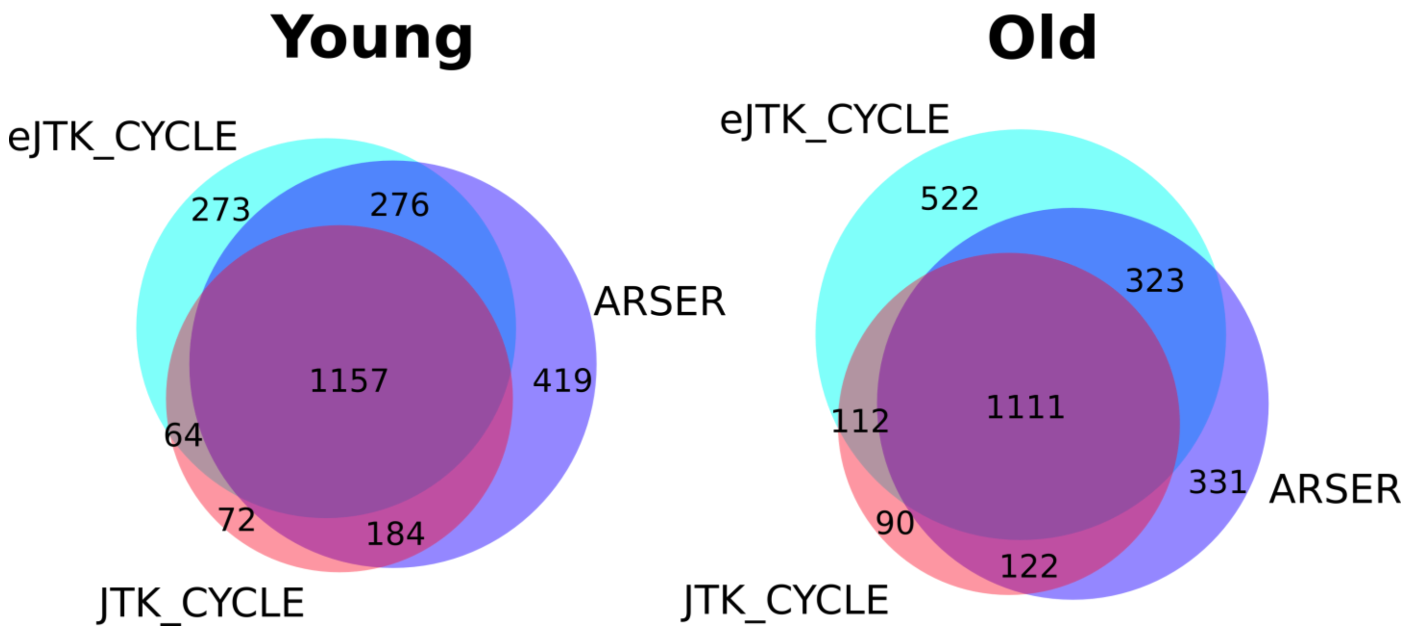

Supplementary Figure 10. Comparison of Three Periodicity Detection Algorithms.

Venn diagrams showing overlap between sets of genes rhythmic at $p \leq 0.05$ in young (left) or old (right) flies according to ARSER, JTK_CYCLE, and eJTK_CYCLE. All genes reported as rhythmic have a max/min fold change ≥ 1.5 and a median FPKM ≥ 1. The p-value used for the eJTK_CYCLE cutoff was the empirical p-value.

	Time Pt.	Rep	Total reads	Total reads after filtering	Uniquely aligned reads	Total aligned reads	Genes with ≥1 FPKM
Young	ZT0	1	34863029	27798961	21520103	22951451	9344
		2	33205307	26321319	19891847	21525853	9342
	ZT4	1	37504482	29902138	23389640	24936441	9379
		2	37051162	29496327	23079532	24737466	9402
	ZT8	1	33495858	26791673	20579297	22118512	9249
		2	38327928	30507618	22423112	24460652	9306
	ZT12	1	37490860	30306789	22904968	24521098	9202
		2	32147690	25606115	20061828	21661687	9224
	ZT16	1	29721164	23798504	18940024	20140185	9269
		2	30806302	24483081	17433120	18823964	9309
	ZT20	1	32526691	25883117	20922373	22022243	9324
		2	34523444	27576263	22330893	23712777	9269
Old	ZT0	1	32223225	26209357	18566978	20262474	9597
		2	33754008	26939858	19348861	21066568	9616
	ZT4	1	32488950	26656035	21026633	22877595	9536
		2	32428446	25979068	20234230	22106834	9568
	ZT8	1	38863347	31402564	23579755	25732874	9511
		2	42404021	33533508	25344632	28093307	9491
	ZT12	1	35787705	28976853	20650698	22618259	9450
		2	38351930	30072633	22430168	24694205	9444
	ZT16	1	31252434	25547287	19512251	21281686	9450
		2	32253980	25902375	18391049	20094376	9568
	ZT20	1	28073110	22850785	17744869	19113621	9539
		2	29377381	23341722	18164021	20087202	9519

Supplementary Table 1. RNA-seq Alignment Statistics.

Read counts were reported by skewer (Jiang et al., 2014) before and after filtering. Alignment counts were reported by TopHat2 (Kim et al., 2013). Transcript abundance was determined with Cuffdiff, which is part of the Cufflinks package (Trapnell et al., 2010). Rep. = biological replicate.

	Genes		Transcripts	
**Sample**	**Adjusted R^2**	**Pearson's**	**Adjusted R^2**	**Pearson's**
**young ZT0**	**0.9994**	**0.9996847**	**0.9994**	**0.9996883**
**young ZT4**	**0.9907**	**0.9953361**	**0.9907**	**0.9953365**
**young ZT8**	**0.9983**	**0.9991708**	**0.9983**	**0.9991724**
**young ZT12**	**0.9842**	**0.9920624**	**0.9842**	**0.9920508**
**young ZT16**	**0.9309**	**0.9648164**	**0.9308**	**0.9647871**
**young ZT20**	**0.9904**	**0.9951922**	**0.9904**	**0.9951833**
**old ZT0**	**0.9692**	**0.9844787**	**0.9697**	**0.9847144**
**old ZT4**	**0.9921**	**0.9960439**	**0.9921**	**0.9960561**
**old ZT8**	**0.9822**	**0.9910556**	**0.9821**	**0.991022**
**old ZT12**	**0.9678**	**0.9837644**	**0.9678**	**0.983764**
**old ZT16**	**0.9318**	**0.9653072**	**0.9319**	**0.9653351**
**old ZT20**	**0.9957**	**0.997858**	**0.9958**	**0.9978744**

Supplementary Table 2. Strong RNA-seq Gene Expression Correlations Among Biological Replicates.

Table of adjusted R^2 and Pearson’s correlation values computed from Cuffdiff-computed gene expression values of transcripts or genes from replicate RNA-seq data sets.

**Statistic**	**Genes**	**Transcripts**
**Mean**	**0.001**	**0.002**
**Standard deviation**	**1.045**	**1.020**
**Gaussian fit μ**	**-0.027**	**-0.037**
**Gaussian fit σ**	**0.927**	**0.834**

Supplementary Table 3. Late-Life Cycler Statistics.

Statistical parameters for S_{DR} distributions and for Gaussian fits to the S_{DR} distributions shown in Supplementary Fig. 4. Gaussian fit equations were computed using Gnuplot’s “fit” function.

Gene ID	Y q-value	O q-value	Y p-value	O p-value	Y Phase	O Phase	Y Median	O Median	Y FC	O FC
XLOC_017514	4.36E-01	8.22E-02	8.48E-02	5.52E-03	5.2	7.0	0.25	0.32	46.44	12.95
XLOC_003947	7.50E-01	5.12E-02	5.50E-01	1.99E-03	9.3	8.9	0.32	17.58	4.19	2.78
XLOC_016308	1.79E-01	4.83E-01	1.63E-02	1.60E-01	0.1	21.1	1.07	2.95	1.57	1.41
XLOC_002540	4.35E-03	5.01E-03	8.64E-05	3.25E-05	8.2	8.1	4.68	2.10	45.40	102.87
XLOC_009560	3.19E-01	1.43E-01	5.17E-02	1.84E-02	18.3	8.3	3.02	3.25	4.65	3.71
XLOC_010325	1.56E-01	6.70E-01	1.32E-02	3.44E-01	20.9	0.2	0.36	0.39	2.81	1.94
XLOC_000178	3.80E-03	2.22E-01	2.47E-05	4.04E-02	0.1	1.6	2.19	2.39	2.75	1.86
XLOC_005505	4.35E-03	7.83E-01	1.02E-04	5.54E-01	21.9	20.4	0.38	0.67	8.93	12.86
XLOC_016359	9.11E-01	1.05E-01	8.11E-01	1.03E-02	19.5	23.4	0.39	3.91	1.51	3.22
XLOC_013970	2.96E-01	3.91E-01	3.74E-02	1.14E-01	13.5	15.7	2.58	1.27	3.34	3.30
XLOC_013967	6.39E-01	2.43E-01	3.58E-01	4.87E-02	7.4	0.9	0.05	0.36	5.63	5.05
XLOC_001839	2.96E-01	5.25E-01	4.03E-02	2.25E-01	0.8	23.4	0.00	3.68	4.91E+06	7.31
XLOC_003260	9.11E-01	1.65E-01	8.35E-01	2.46E-02	16.4	20.0	0.15	0.19	3.54	4.60
XLOC_016491	2.96E-01	5.04E-01	3.27E-02	2.06E-01	21.7	9.4	0.00	0.00	3.49E+06	1.77E+06
XLOC_011167	3.75E-02	2.43E-01	1.95E-03	5.51E-02	20.3	4.3	0.04	3.46	5.09E+05	1.72
XLOC_003905	6.28E-01	1.30E-01	3.14E-01	1.43E-02	15.8	23.9	0.79	1.49	3.49	3.62
XLOC_000088	5.60E-01	1.82E-01	1.69E-01	2.83E-02	3.4	3.1	0.14	0.21	6.17	4.25
XLOC_019389	4.34E-02	2.22E-01	2.54E-03	4.17E-02	22.3	0.1	12.23	11.61	1.46	1.55
XLOC_004292	9.02E-01	1.98E-01	7.77E-01	3.25E-02	2.0	21.5	0.63	0.53	4.73	6.24
XLOC_000146	5.60E-01	1.43E-01	1.80E-01	1.71E-02	21.4	22.1	2.15	1.66	1.99	1.90
XLOC_015813	3.19E-01	1.05E-01	4.69E-02	9.57E-03	12.4	8.9	2.31	1.30	3.17	5.97
XLOC_018922	2.96E-01	2.22E-01	3.15E-02	3.89E-02	19.6	19.6	0.10	0.28	5.26E+06	7.37
XLOC_016378	4.53E-01	1.04E-01	1.03E-01	8.13E-03	1.6	1.1	1.17	1.07	2.41	2.20
XLOC_016358	6.05E-01	1.72E-02	2.20E-01	3.07E-04	23.0	0.6	0.58	2.95	2.35	2.26
XLOC_017264	1.53E-01	2.52E-01	1.09E-02	6.22E-02	19.9	18.8	0.68	1.14	1.93	3.07
XLOC_017687	3.10E-02	8.22E-02	1.41E-03	4.82E-03	1.9	3.2	5.02	11.69	1.67	2.19
XLOC_011153	6.66E-03	1.72E-02	2.16E-04	3.35E-04	8.1	9.9	0.56	1.50	10.25	3.11
XLOC_016356	5.55E-01	3.39E-02	1.45E-01	1.10E-03	0.9	2.5	0.57	1.87	1.69	2.31
XLOC_001630	2.96E-01	7.67E-01	3.48E-02	5.11E-01	18.0	21.0	0.93	1.60	1.51	2.05
XLOC_005560	6.34E-01	1.05E-01	3.29E-01	9.46E-03	12.5	18.3	0.60	0.73	1.31E+07	1.18E+03
XLOC_013966	6.26E-01	1.50E-01	2.62E-01	2.05E-02	15.8	20.1	0.37	1.81	1.93	1.59
XLOC_002709	6.39E-01	8.22E-02	3.61E-01	4.35E-03	16.0	23.9	0.57	0.29	12.66	7.79E+06
XLOC_009520	9.02E-01	1.43E-01	7.71E-01	1.86E-02	20.8	23.7	2.14	1.80	1.43	1.75
XLOC_005507	3.19E-01	5.42E-01	4.81E-02	2.60E-01	7.8	8.8	2.10	7.99	1.48	1.35
XLOC_012909	1.35E-01	4.95E-01	8.76E-03	1.67E-01	9.4	11.9	0.49	0.15	8.55E+04	6.92E+04
XLOC_011166	1.56E-01	1.30E-01	1.28E-02	1.37E-02	18.3	16.5	28.74	29.16	1.93	1.83
XLOC_003903	6.00E-01	2.43E-01	2.11E-01	4.98E-02	7.5	11.7	1.85	4.11	1.58	1.56
XLOC_000172	4.47E-01	1.65E-01	9.00E-02	2.46E-02	4.2	5.1	1.85	6.61	2.61	2.27
XLOC_003867	7.96E-01	8.22E-02	6.04E-01	5.87E-03	2.6	22.8	1.97	2.68	2.37	2.26
XLOC_009032	1.99E-02	1.98E-01	7.73E-04	3.34E-02	7.4	6.8	3.38	5.91	2.38	1.76
XLOC_016319	8.39E-01	6.32E-02	6.71E-01	2.87E-03	0.2	22.0	0.84	0.99	1.87	2.29
XLOC_009531	2.96E-01	5.04E-01	3.88E-02	1.90E-01	6.1	10.4	0.47	0.93	2.94	2.17
XLOC_000890	4.35E-03	1.79E-02	1.13E-04	4.65E-04	7.8	7.5	3.36	1.17	40.93	146.39
XLOC_000007	2.96E-01	8.67E-01	3.27E-02	7.45E-01	20.6	22.7	0.28	0.41	2.56	3.32

Supplementary Table 4. ARSER Output for Rhythmic Putative Novel Genes.

ARSER-reported p-values, q-values (BH-corrected p-values), and phases for novel genes rhythmic in young (Y) or old (O) flies with p-value ≤ 0.05. The last two columns represent expression max/min fold change (FC) in young or old flies, computed after adding a pseudo-count (lowest nonzero expression value) to the max and min values to avoid divide by zero errors.

Gene Symbol (Best Isoform)	Gene Product	Phase	S_{DR} P-value	S_{DR} BH FDR	Best Human/Mouse Ortholog	Stress-Induced Upregulation			
						1	2	3	4
ImpL3-RA	lactate dehydrogenase	12	5.33E-15	5.12E-11	LDHA/Ldha	O	O	H, O	P, H, T
Hsp22-RB	chaperone	12	3.08E-13	1.48E-09	HSPB8/Hspb8	O	O	HS, H, O	P, H, T
Hsp70Bb-RA[a]	heat shock protein 70	7	1.06E-07	2.54E-04	HSPA1A/Hspa1l	O	O	HS, H, O, IR	P, H, T
Hsp26-RA	chaperone	9	2.38E-07	4.58E-04	HSPB1/Cryaa		O	HS, H, O	P
bnl-RC	cytokine	14	3.03E-07	4.85E-04	FGF20/Fgf20	O		H	
CR43264-RA	non-coding RNA	20	4.28E-07	5.87E-04		NA	NA	NA	NA
CG7130-RA	chaperone	13	1.99E-06	2.13E-03	DNAJB8/Dnajc5g	O	O	HS, O, IR	P, T
CG9717-RA	sulfate transporter	22	2.39E-06	2.29E-03	SLC26A11/Slc26a11		NA		
CG5002-RA	sulfate transporter	12	2.77E-06	2.42E-03	SLC26A11/Slc26a11				
CG6428-RA	asparaginase	12	8.48E-06	6.05E-03	ASPG/Aspg		O	HS, H, O, IR	P
Jhl-21-RB	amino acid transporter	10	8.81E-06	6.05E-03	SLC7A5/Slc7a5				
Amnionless-RB	membrane protein	8	2.42E-05	1.19E-02	AMN/Amn			H	
Or46a-RB	olfactory receptor	8	2.48E-05	1.19E-02			NA		
CG14545-RA	unknown	22	2.99E-05	1.37E-02				IR	P, H, T
Hsp70Bc-RA[a]	heat shock protein 70	10	3.33E-05	1.46E-02	HSPA1A/Hspa1l	O	O	HS, H, O, IR	P, H, T
a-RD	PDZ protein	8	3.91E-05	1.63E-02	PDZD2/Pdzd2	O		HS, IR	
Mmp1-RC	metallopeptidase	10	5.84E-05	2.34E-02	MMP14/Mmp14			O, IR	
CG14636-RA	unknown	22	8.17E-05	3.04E-02			NA		
CG15784-RA	unknown	12	8.35E-05	3.04E-02	HRG/Hrg	O	O	HS, H, O, IR	P, H, T
CG10383-RA	ester hydrolase	8	1.19E-04	3.68E-02	SERAC1/Serac1		O	HS, H, O, IR	P, H
Skp2-RE	ubiquitin ligase	13	1.30E-04	3.89E-02	SKP2/Skp2				
CG18869-RB	glucosyltransferase	6	1.62E-04	4.51E-02	UGT2B11/Ugt2b38			HS, H, IR	
Lip3-RA	lipase	2	1.69E-04	4.51E-02	LIPF/Lipa				
CG43055-RA	lectin	10	1.81E-04	4.71E-02	MBL2/Cd209c	NA	NA	NA	NA
CG17121-RA	oxidoreductase	8	1.95E-04	4.80E-02	DHRS3/Dhrs3			H, IR	

[a] Shared microarray probes

Supplementary Table 5. LLC Characterization.

Top LLC genes. Best isoforms were identified by assigning an S_{DR} to individual transcripts as for genes; the isoform with the best S_{DR} that satisfied rhythmicity criteria was selected. The Phase column shows ARSER-reported phases rounded to the nearest integer. Gene orthologs were predicted by DIOPT. Gene products are from FlyBase. Abbreviations: O = O_2 (hyperoxia); H = H_2O_2; HS = heat shock; P = paraquat, oxidative stress inducer; T = tunamycin, ER stress inducer; IR = ionizing radiation; NA = not present in probe set or not reported; S_{DR} = differential rhythmicity score; BH FDR = Benjamini-Hochberg false discovery rate. Gene upregulation by stress type was reported for the following studies: 1 = Gruenewald, et al. (2009); 2 = Landis, et al. (2004); 3 = Landis, et al. (2012); 4 = Girardot, et al. (2004). Full references are provided at the end of the Supplementary Data document.

Target	Forward (5' to 3')	Reverse (5' to 3')
DCP2	**CCAAGGGCAAGATCAATGAG**	**GCATCGATTAGGTCCGTGAT**
Clk	**CCTTACGAAGGGCCAACAGT**	**ACCTCGGCATAGCTGACAAC**
tim	**GTGCTTCTGCTGAGGCGTTTCAAT**	**GGCGAATGGTTTGACATCCACCAA**
per	**GCGCGACCGGACAACTAC**	**GTGTGGCGTATGGCGAACT**
ImpL3	**CGTTTGGTCTGGAGTGAACA**	**GCAGCTCGTTCCACTTCTCT**
bnl	**GGAACCCAGGACGAGAATAG**	**GCGTCCATGCAGAGATACAG**
Hsp22	**GTCAACAAGGATGGCTACAA**	**CACCAGGACAACGCTCTC**
CG7130	**TCCAGTTCTTCAACCGGAAA**	**AGGTACCCGAGATTGCTTCA**
CG15784	**ATGGCCGTCGACTAGAGAGA**	**AACTGGAGCTGGAACTGGAA**
crescendo	**AACAAGAACTGGAAGAAGTTAGGG**	**TTATAGCGTAGTCTTTGTGACTTGC**

Supplementary Table 6. Primer Sequences Used for qRT-PCR.

	Young	Old	Both	Overlap (Young)	Overlap (Old)
**ARSER**	**2036**	**1887**	**922**	**45.28%**	**48.86%**
**JTK_CYCLE**	**1477**	**1435**	**572**	**38.73%**	**39.86%**
**eJTK_CYCLE**	**1770**	**2068**	**790**	**44.63%**	**38.20%**

Supplementary Table 7. Rhythmic Gene Counts According to Three Rhythmicity Detection Methods.

Table of counts for genes rhythmic in heads of young flies, old flies, or both according to ARSER, JTK_CYCLE, and eJTK_CYCLE at p-value ≤ 0.05. All genes reported as rhythmic have a max/min fold change ≥ 1.5 and a median FPKM ≥ 1. The p-value used for the eJTK_CYCLE cutoff was the empirical p-value. Percentage overlap represents the proportion of genes rhythmic in both young and old divided by the number genes rhythmic in either young or old.

Supplementary References

1. Gruenewald, C., Botella, J.A., Bayersdorfer, F., Navarro, J.A., and Schneuwly, S. (2009). Hyperoxia-induced neurodegeneration as a tool to identify neuroprotective genes in Drosophila melanogaster. Free Radical Biol Med *46*, 1668-1676.

2. Landis, G.N., Abdueva, D., Skvortsov, D., Yang, J., Rabin, B.E., Carrick, J., Tavare, S., and Tower, J. (2004).Similar gene expression patterns characterize aging and oxidative stress in Drosophila melanogaster. Proc Natl Acad Sci U S A *101*, 7663-7668.

3. Landis, G., Shen, J., and Tower, J. (2012). Gene expression changes in response to aging compared to heat stress, oxidative stress and ionizing radiation in Drosophila melanogaster. Aging (Albany NY) *4*, 768-789.

4. Girardot, F., Monnier, V., and Tricoire, H. (2004). Genome wide analysis of common and specific stress responses in adult *Drosophila melanogaster*. BMC Genomics *5, 74* DOI: 10.1186/1471-2164-5-74.
